# Association between hippocampal subfield volumes and working memory in middle-aged and older adult cancer patients

**DOI:** 10.3389/fnhum.2025.1681302

**Published:** 2025-12-15

**Authors:** Ran Bi, Yue Liu, Qiaoyang Zhang, Guanzhong Dong, Ke Xu, Yin Cao

**Affiliations:** 1 Department of Psychology, Third Affiliated Hospital of Nanjing Medical University, Changzhou, China; 2 Changzhou Medical Center, Nanjing Medical University, Changzhou, China

**Keywords:** cancer, working memory, hippocampal subfields, cancer-related cognitive impairment, magnetic resonance imaging

## Abstract

**Background:**

Cancer-related cognitive impairment (CRCI) is relatively common among middle-aged and older adult cancer patients, with working memory deficits being particularly prominent. However, the underlying structural basis of hippocampal subregions remains unclear. This study aimed to investigate differences in hippocampal subfield volumes and working memory function between cancer patients and healthy controls, as well as to analyze the correlation between hippocampal structural alterations and working memory impairment.

**Methods:**

The cohort comprised 51 cancer patients and 45 healthy controls. All participants underwent 3D-T1 structural MRI scans and cognitive assessments. Hippocampal subfields were automatically segmented using FreeSurfer 7.4, and their volumes were calculated. Group differences in cognitive test scores were compared. After controlling for total intracranial volume (TIV), analysis of covariance (ANCOVA) was performed to examine differences in hippocampal subfield volumes between groups. Spearman correlation analysis was undertaken to assess the relationship between hippocampal subfield volumes and cognitive test scores in patients with cancer.

**Results:**

Compared with healthy controls, cancer patients exhibited significantly lower scores in the digit span test (DST) total score (*U* = 716.50, *p* = 0.001) and digit span forward (DSF) subtest (*U* = 738.50, *p* = 0.002). Hippocampal subfield analysis revealed significant volume reductions in the cancer group, particularly in CA3 (*F* = 8.141, *p* = 0.005) and CA4 (*F* = 6.770, *p* = 0.011). Correlation analysis demonstrated that the volumes of the hippocampal head (*r* = 0.410, *p* = 0.003) and hippocampal molecular layer (*r* = 0.389, *p* = 0.005) were positively associated with DST scores in cancer patients.

**Conclusion:**

Cancer patients exhibit working memory impairment and hippocampal subfield atrophy. The significant correlation between the volumes of the hippocampal head and molecular layer with working memory performance suggests that these regions may play a critical role in cancer-related cognitive dysfunction.

## 1 Introduction

With advancements in cancer treatment, the survival of middle-aged and older adult cancer patients has significantly increased. Concurrently, treatment-related neurocognitive sequelae have garnered increasing attentio (Oldacres et al., 2023). Previous studies indicate that the prevalence of cancer-related cognitive impairment (CRCI) ranges between 21 and 25% (Dijkshoorn et al., 2021). This cognitive impairment has been confirmed to reduce patients’ quality of life significantly (Kerstens et al., 2023). The clinical manifestations of cancer-related cognitive impairment (CRCI) exhibit multidimensional characteristics and can affect multiple cognitive domains over the long term. A 20-year longitudinal study demonstrated that breast cancer survivors with a history of chemotherapy exhibited significantly lower performance in key cognitive functions such as processing speed, executive function, and verbal memory compared to healthy controls, even two decades after treatment (Koppelmans et al., 2012). It is particularly noteworthy that a similar pattern of broad cognitive decline has been observed in patients with localized colorectal cancer. Research by Vardy et al. (2014) confirmed that this patient population shows decline across multiple dimensions—including verbal learning and memory, visual learning and memory, processing speed, attention, and working memory—relative to healthy controls.

Among these deficits, impairments in memory function often lead to more profound clinical consequences. These include reduced treatment adherence, impaired occupational capacity, and a diminished quality of daily life (McDougall et al., 2014; Alatawi et al., 2021; Oliva et al., 2024). Through a systematic literature review, Rodrigues et al. (2023) identified working memory (WM) deficits as the primary manifestation of memory impairment in cancer patients. Based on the classic multi-component working memory model proposed by Baddeley and Hitch, working memory is defined as a capacity-limited system responsible for temporary processing and storage of information, primarily comprising three core subsystems: the phonological loop, visuospatial sketchpad, and central executive. Numerous empirical studies have validated impairments in various subsystems of working memory in cancer patients from different perspectives. A study by Goktas Aydin et al. (2025) using Trail Making Test B and the Stroop Test found that colorectal cancer patients (particularly those with stage IV) demonstrated significantly poorer performance in task switching and inhibitory control, suggesting impaired executive function and indirectly reflecting compromised central executive system functioning. Another study focusing on gynecological patients revealed that the patient group scored significantly lower than healthy controls on the digit span test, indicating potential impairment of the phonological loop (Zeng et al., 2019). Furthermore, research by Siegwart et al. (2021) employing the Block Recall Test to assess survivors of childhood non-CNS cancers found poorer performance in visual working memory tasks, suggesting a declining trend in visuospatial sketchpad function.

The hippocampus, a core structure within the medial temporal lobe, plays a crucial role in functions such as memory encoding, learning, and emotional regulation. Research shows that pathological changes in the hippocampus are closely associated with various diseases, including Alzheimer’s disease, schizophrenia, and multiple sclerosis (Piskorowski and Chevaleyre, 2023). Notably, patients with cancer-related cognitive impairment exhibit reduced hippocampal volume (Daniel et al., 2024). The hippocampus is not a functionally homogeneous structure, exhibiting significant heterogeneity in anatomical connectivity, molecular expression, and function among its internal subfields. The classic trisynaptic circuit elucidates the fundamental division of labor within hippocampal subfields: the dentate gyrus is responsible for “pattern separation,” preventing memory interference; the CA3 region achieves “pattern completion” through its robust recurrent connections, enabling recall of complete memories from partial cues; and the CA1 region integrates information and ultimately generates output signals from the hippocampus to other brain regions (Neves et al., 2008). Additionally, research by Hitti and Siegelbaum (2014) indicates that the CA2 subfield of the hippocampus is a critical brain area for social memory. This anatomical and functional specificity motivates a more granular investigation beyond total hippocampal volume.

Studies conducted in elderly populations have investigated the relationship between hippocampal subfield volumes and working memory. Their correlation analyses revealed that working memory performance was associated with the left hippocampal head of CA1, head of CA3, head and body of CA4, head of the molecular layer, as well as head and body of GC-ML-DG. Simultaneously, correlations were also observed with the right hippocampal head of CA1, head of CA3, head of CA4, head of GC-ML-DG, head of the molecular layer, HATA, and head of the presubiculum (Freibergs et al., 2025). However, whether a similar association exists in cancer patients remains largely unexplored.

In terms of structural neuroimaging methods, high-resolution 3D-T1-weighted MRI has become a fundamental technical basis for quantitative analysis of hippocampal subfield volumes. This sequence provides excellent gray-white matter contrast and whole-brain coverage, enabling precise segmentation and volumetric measurement of hippocampal subfields, thereby offering reliable neuroimaging evidence for investigating their associations with cognitive functions (Wisse et al., 2017).

This study aims to investigate the specific neural mechanisms underlying working memory (WM) impairment in cancer-related cognitive impairment (CRCI). We employed the digit span test for assessment. This test consists of two components: the forward span, which primarily reflects the storage capacity of the phonological loop, and the backward span, which requires the involvement of the central executive in manipulating information. To further examine the integrity of the central executive, we also included the verbal fluency test (VFT) as a reference. Based on this, we propose the following hypotheses: (1) Compared with the healthy control (HC) group, cancer patients will demonstrate significantly poorer performance on working memory tasks; (2) The patient group will exhibit volume reduction in specific subregions of the hippocampus; and (3) We further hypothesize that volume changes in key hippocampal subregions will be significantly correlated with the severity of working memory impairment.

## 2 Materials and methods

### 2.1 Participants

This study consecutively enrolled 51 middle-aged and older adult cancer patients admitted to the Department of Oncology at Changzhou Second People’s Hospital between November 2023 and June 2024. Forty-five healthy controls (HC) were recruited from the community. All patients completed emotional assessments, cognitive assessments, and brain MRI scans. The demographic and clinical characteristics of all participants are summarized in Table 1. Inclusion criteria for the cancer group: (1) Pathologically confirmed cancer diagnosis; (2) Age ≥45 years; (3) Right-handedness; (4) Somatic diseases effectively controlled; (5) Provision of written informed consent. Exclusion criteria: (1) Brain metastasis; (2) History of tumor resection within the past 2 weeks; (3) Organic brain diseases, substance-induced mental disorders (including chemotherapy and radiotherapy), or psychiatric disorders; (4) Comorbid abuse of substances such as tobacco or alcohol; (5) Contraindications for MRI examination (e.g., implanted cardiac pacemaker, stent, intraocular metal foreign body; high fever, fixed dentures, metal implants, history of intracranial metal foreign body splinters, claustrophobia). This study was conducted in accordance with the principles of the Declaration of Helsinki. This study was reviewed and approved by the Medical Ethics Committee of the Third Affiliated Hospital of Nanjing Medical University [Ethics Approval No. (2021)YLJSC003]. All participants provided written informed consent.

**Table 1:** Comparison of general clinical characteristics between groups.

Variable	Cancer group (*n* = 51)	Healthy group (*n* = 45)	Test value	*p*
Gender, *n* (%)			1.865^a^	0.172
Male	32 (62.7)	22 (48.9)		
Female	19 (37.3)	23 (51.1)		
Age, mean ± SD	66.3 ± 8.8	63.8 ± 8.5	2.045^b^	0.156
Education, *n* (%)			Fisher	0.223
Illiterate	2 (3.9)	5 (11.1)		
Primary school	18 (35.3)	17 (37.8)		
Junior high	29 (56.9)	18 (40)		
High school/Technical school	2 (3.9)	3 (6.7)		
College or above	0 (0)	2 (4.4)		
Hypertension, *n* (%)	29 (56.9)	27 (60)	0.097^a^	0.756
Diabetes, *n* (%)	41 (80.4)	28 (62.2)	Fisher	0.068

a *χ*^2^ value for chi-square test.

b *F* value for independent samples *t*-test.

### 2.2 Data collection

#### 2.2.1 General information

A self-designed general information questionnaire was used to collect socio-demographic data, including sex, age, education level, and information on hypertension and diabetes.

#### 2.2.2 Cognitive assessment

The verbal fluency test (VFT) and digit span test (DST) were selected as objective assessments of cognitive function. The VFT focuses on linguistic executive function, requiring participants to name as many words as possible beginning with the “(Fa)” within 1 min. The final VFT score was the number of words generated, with higher scores indicating better cognitive function. The DST assesses working memory and attention, comprising the digit span forward (DSF) and digit span backward (DSB) tests. Testing procedure: DSF and DSB were administered separately. The examiner pronounced digits at a rate of one digit per second, with intervals between digits, thereby avoiding the grouping of longer sequences. DSF method: All participants started with the first item (3 digits) of the first trial. If answered correctly, they proceeded to the next item. If incorrect, a second trial of the same item was administered. Testing ceased if both trials of an item failed. Scoring: The highest number of digits successfully repeated constituted the score (e.g., successfully repeating seven digits scored 7 points, without accumulating lower scores). DSB method: A demonstration was given first (e.g., “7-1-9” recalled as “9-1-7″). If correct, testing began with the first 3-digit item. If the participant provided an incorrect answer or misunderstood, the correct answer was provided, and another example was given. If successful with the example, testing proceeded with three digits. If unsuccessful with the example, testing began with two digits. If the participant passed the example but failed both trials of the first 3-digit item, testing returned to 2 digits and concluded. Scoring was identical to DSF. The final DST score was the sum of the DSF and DSB scores, with higher scores indicating better cognitive function.

### 2.3 MRI data acquisition and processing

T1-weighted structural MRI scans were acquired for all enrolled participants. All MRI scans were performed using a GE Discovery MR750W 3.0 T MRI scanner at the Yanghu Campus of Changzhou Second People’s Hospital. 3D-T1 scan parameters: T1-weighted 3D-MPRAGE sequence. TR = 1,900 ms, TE = 2.48 ms, TI = 900 ms, flip angle = 9°, slices = 176, slice thickness = 1 mm, slice gap = 0 mm, matrix = 256 × 256, FOV = 250 mm × 250 mm. Scan time: 4 min 18 s. Images were converted to NifTI format using MRIcron software and imported into FreeSurfer version 7.4.1. Preprocessing, including motion correction, normalization, skull stripping, and tissue segmentation, was performed using the recon-all command. Following whole-brain structural segmentation, the segmentHA_T1.sh command was used for automated segmentation of hippocampal subfields, yielding 19 subfields per hemisphere: cornu ammonis 1 (CA1) head, CA1 body, CA3 head, CA3 body, CA4 head, CA4 body, subiculum head, subiculum body, parasubiculum, presubiculum head, presubiculum body, molecular layer head, molecular layer body, GC-ML-DG head (granule cell and molecular layer of the dentate gyrus), GC-ML-DG body, hippocampal fissure, fimbria, hippocampal-amygdaloid transition area (HATA), and hippocampal tail (Table 2). Segmentation quality was visually inspected to ensure accuracy. Intracranial volume (ICV), total hippocampal volume, and subfield volume data were extracted for each participant. To balance the mitigation of multiple comparison risks with the retention of crucial anatomical information, our analysis did not encompass all 19 hippocampal subfields segmented by FreeSurfer. Instead, we focused on core gray matter subfields, selected based on criteria integrating theoretical rationale and data robustness: (1) Priority was given to subfields previously discussed in the Introduction as functionally relevant to working memory. (2) Subfields with clearly defined anatomy and well-established functions were chosen. (3) Subfields with ambiguous functions, such as the hippocampal-cortical transition zones, and white matter structures primarily composed of fiber tracts (e.g., the fimbria), were excluded. The specific subfields ultimately included in our analysis were: CA1, CA3, CA4, the hippocampal molecular layer (ML), the granule cell and molecular layer of the dentate gyrus (GC-ML-DG), the subiculum, the hippocampal tail, the hippocampal body, and the hippocampal head.

**Table 2:** Hippocampal subfield segmentation (HBT protocol).

Hippocampal subregion name	Associated macroregion
Parasubiculum	Hippocampal-head
Presubiculum-head	
Subiculum-head	
CA1-head	
CA3-head	
CA4-head	
GC-ML-DG-head	
Molecular_layer_HP-head	
HATA	
Presubiculum-body	Hippocampal-body
Subiculum-body	
CA1-body	
CA3-body	
CA4-body	
GC-ML-DG-body	
Molecular_layer_HP-body	
Fimbria hippocampi	
Hippocampal tail	Hippocampal-tail
Hippocampal-fissure	Hippocampal-fissure

### 2.4 Statistical analysis

All statistical analyses were performed using SPSS software (version 27.0). The normality of continuous variables was assessed using the skewness–kurtosis test. Normally distributed variables are presented as mean ± standard deviation, whereas non-normally distributed variables are reported as median and interquartile range [M (Q1, Q3)]. Categorical variables are summarized as frequencies and percentages. For between-group comparisons of demographic and clinical baseline characteristics, the following procedures were applied: after evaluating the homogeneity of variance using Levene’s test, continuous variables that met both normality and homogeneity assumptions were analyzed using independent-samples *t*-tests; otherwise, the Mann–Whitney *U* test was used. Categorical variables were compared using the chi-square test.

Between-group differences in hippocampal subfield volumes were assessed using analysis of covariance (ANCOVA), with group entered as a fixed factor and total intracranial volume (TIV) included as a covariate to control for potential bias arising from differences in total brain volume.

To specifically investigate the relationship between brain structure and clinical symptoms in the disease state, exploratory Spearman correlation analyses were conducted exclusively within the cancer patient group to examine associations between cognitive performance and hippocampal subfield volumes. This approach was chosen to address a core clinical question: whether the severity of cognitive dysfunction in the patient cohort is related to the degree of structural damage in specific brain regions. To comprehensively explore potential trends, all hippocampal subfields and cognitive scale scores that showed nominal significance (uncorrected *p* < 0.05) in the initial between-group analyses were included in this correlation analysis.

To control for the increased risk of false positives due to multiple comparisons, all statistical tests performed in this study—including between-group comparisons of cognitive scale scores, hippocampal subfield volumes, and correlation analyses—were corrected using the Benjamini–Hochberg false discovery rate (FDR) procedure. The significance threshold was set at FDR-corrected *p* < 0.05.

## 3 Results

### 3.1 Comparison of general clinical data

Ninety-six middle-aged and older participants were included (54 males, 42 females; age range, 45–82 years; mean, 65.1 ± 8.7 years), comprising 51 cancer patients and 45 healthy controls. There were no significant differences between the two groups in terms of age, sex, education level, proportion with hypertension, or proportion with diabetes (*p* > 0.05). Table 1 summarizes the demographic and clinical characteristics of the participants.

### 3.2 Comparison of cognitive function parameters

**Figure 1:**
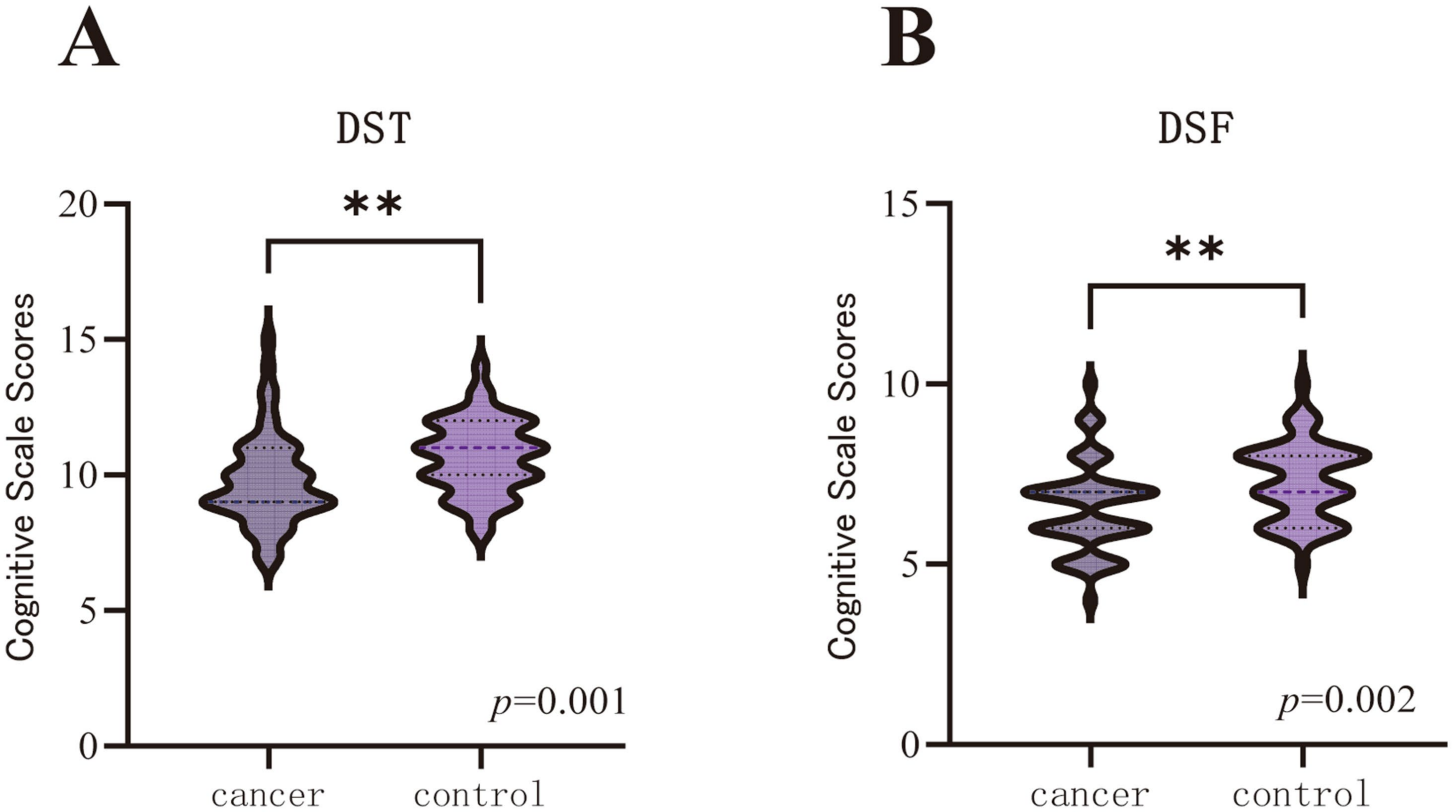
Comparison of cognitive scale scores between cancer and healthy control groups. **(A)** DST scores. **(B)** DSF scores. (DST, digit span total; DSF, digit span forward. The horizontal dashed line indicates the median, and the dotted lines represent the first and third quartiles. ^**^Indicates *p* < 0.05 after FDR correction).

Mann–Whitney *U* tests reveal that the cancer group performed significantly lower than the healthy control group in specific cognitive domains (Table 3 and Figure 1). The cancer group showed significantly reduced scores on the DST (*U* = 716.50, *p* = 0.001) and DSF (*U* = 738.50, *p* = 0.002). The differences in DSB and VFT scores did not reach statistical significance.

**Table 3:** Comparison of cognitive scale scores between groups.

Variable	Cancer group [M (Q1, Q3)]	Healthy group [M (Q1, Q3)]	*U*	*p*
VFT	3 (3, 4)	4 (3, 5)	1012.50	0.307
DST	9 (9, 11)	11 (10, 12)	716.50	0.001^a^
DSF	7 (6, 7)	7 (6, 8)	738.50	0.002^a^
DSB	3 (3, 4)	3 (3, 4)	976.00	0.164

a Indicates *p* < 0.05 after FDR correction.

### 3.3 Comparison of hippocampal subfield volumes

**Figure 2:**
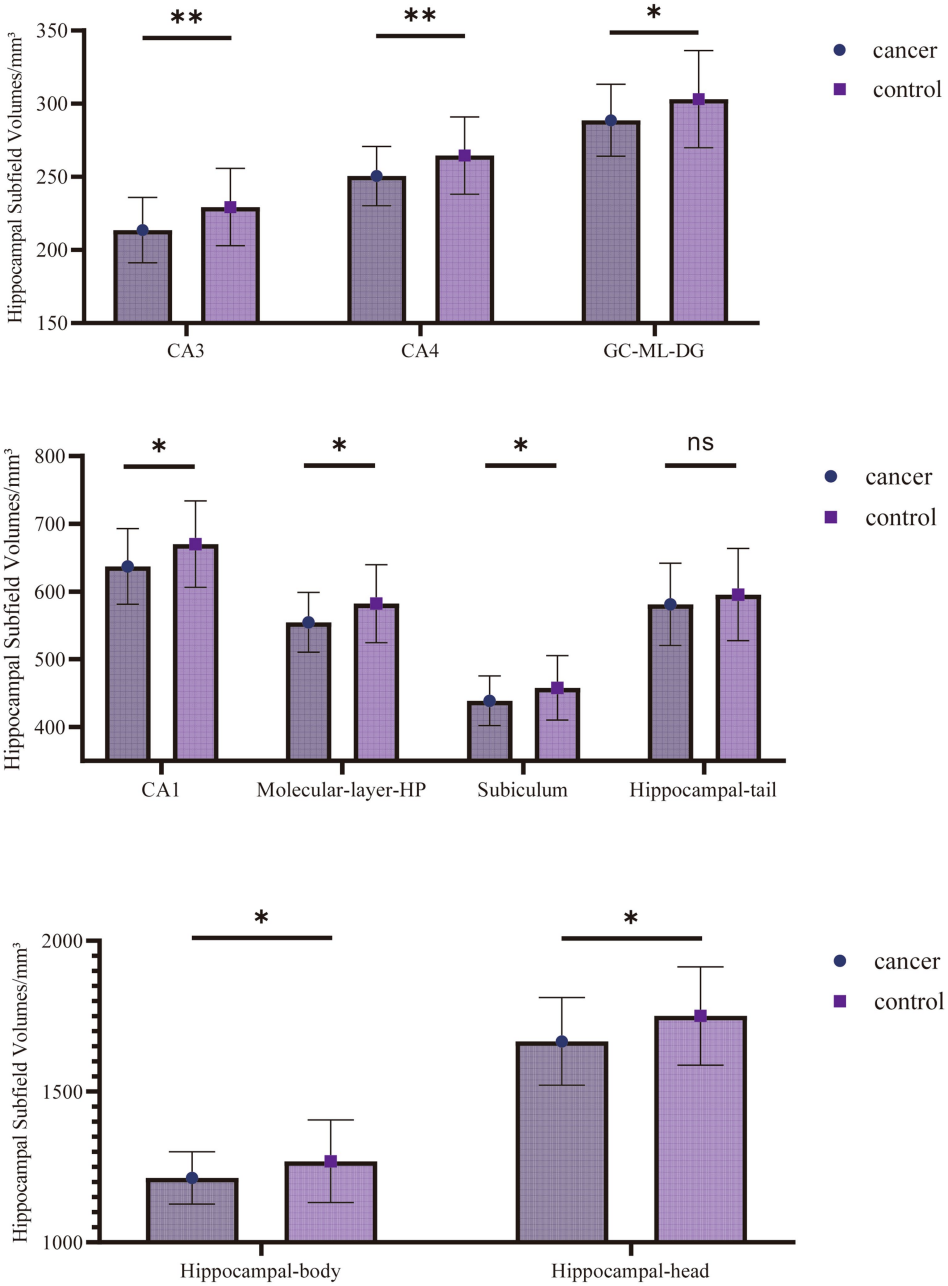
Comparison of hippocampal subfield volumes between cancer and healthy control groups. (^*^Indicates *p* < 0.05 for group comparison. ^**^Indicates *p* < 0.05 after FDR correction).

Analysis of covariance (ANCOVA), with intracranial volume (ICV) as a covariate, reveals significant differences between the cancer and healthy control groups in several hippocampal subfield volumes (Table 4 and Figure 2). Volume reductions are most pronounced in the CA3 (*F* = 8.141, *p* = 0.005) and CA4 (*F* = 6.770, *p* = 0.011) subfields, and remained significant after FDR correction (*p* < 0.05). Significant differences (*p* < 0.05) are validated for the other subfields CA1 (*F* = 5.490, *p* = 0.021), hippocampal molecular layer (ML) (*F* = 5.612, *p* = 0.020), GC-ML-DG (*F* = 4.637, *p* = 0.034), subiculum (*F* = 3.974, *p* = 0.049), hippocampal head (*F* = 5.618, *p* = 0.020), and hippocampal body (*F* = 4.729, *p* = 0.032); however, these did not survive FDR correction. No significant group difference was found for hippocampal tail volume (*F* = 0.591, *p* = 0.444).

**Table 4:** Comparison of hippocampal subfield volumes between groups.

Hippocampal subregion	Cancer group (mean ± SD, mm^3^)	Healthy group (mean ± SD, mm^3^)	*F*	*p*
CA1	637.13 ± 55.95	670.07 ± 63.76	5.490	0.021^a^
CA3	213.64 ± 22.35	229.30 ± 26.37	8.141	0.005^b^
CA4	250.55 ± 20.27	264.50 ± 26.49	6.770	0.011^b^
Molecular-layer	554.58 ± 44.16	582.26 ± 57.61	5.612	0.020^a^
GC-ML-DG	288.64 ± 24.64	303.02 ± 33.16	4.637	0.034^a^
Subiculum	438.84 ± 36.56	457.89 ± 47.69	3.974	0.049^a^
Hippocampal tail	581.24 ± 60.69	595.59 ± 68.19	0.591	0.444
Hippocampal body	1213.80 ± 86.58	1268.91 ± 137.11	4.729	0.032^a^
Hippocampal head	1666.41 ± 145.45	1750.97 ± 162.95	5.618	0.020^a^

a Indicates *p* < 0.05 for comparison between cancer and healthy control groups.

b Indicates *p* < 0.05 after FDR correction.

### 3.4 Correlation analysis between hippocampal subfield volumes and cognitive scale scores in the cancer group

**Figure 3:**
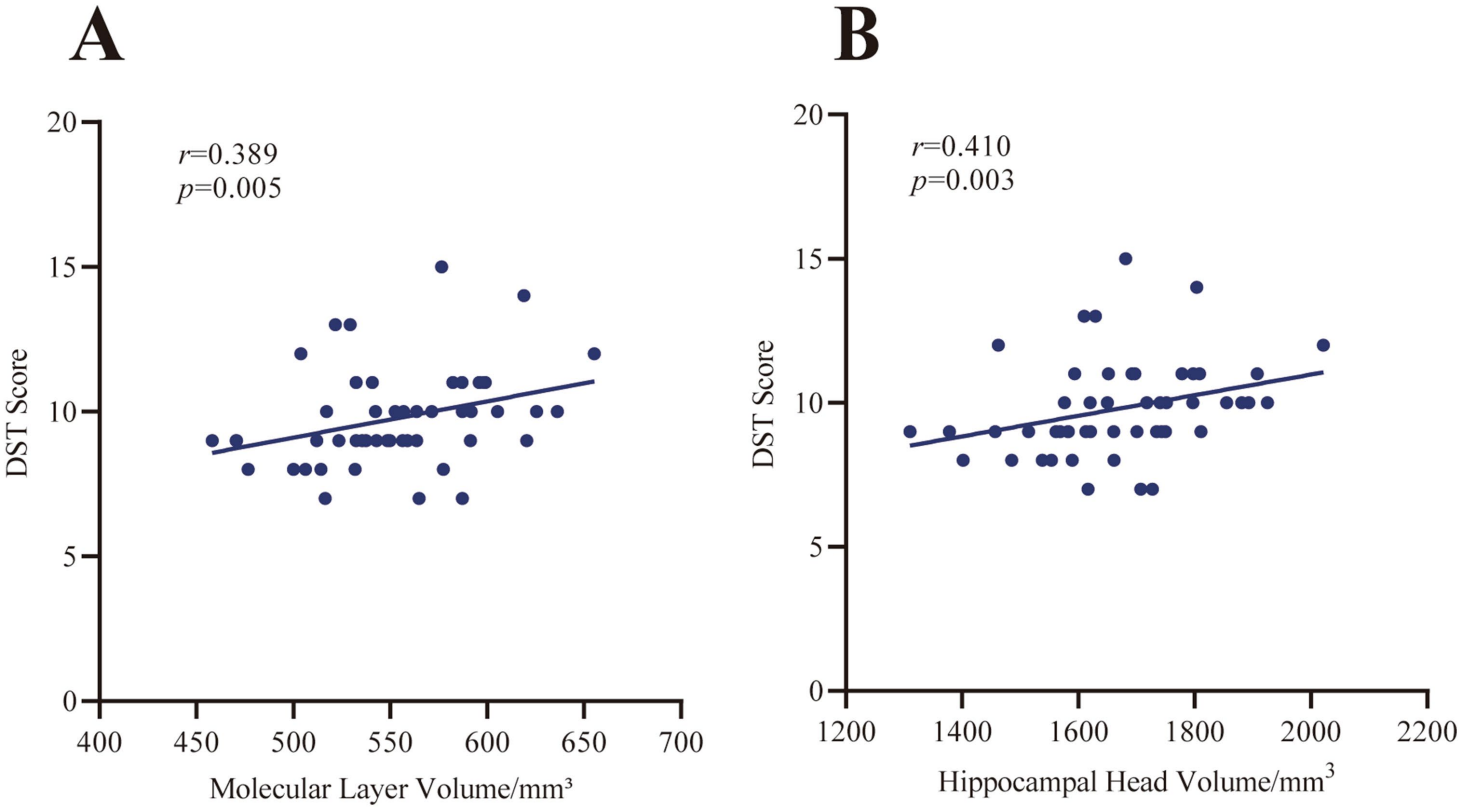
Correlation between DST scores and hippocampal subfield volumes in the cancer group. **(A)** DST score vs. molecular layer volume. **(B)** DST score vs. hippocampal head volume.

Spearman correlation analysis reveals significant positive correlations between the volumes of some hippocampal subfields and DST and DSF scores (Table 5 and Figure 3). The volumes of the hippocampal molecular layer (DST: *r* = 0.389, *p* = 0.005; DSF: *r* = 0.358, *p* = 0.010) and hippocampal head (DST: *r* = 0.410, *p* = 0.003; DSF: *r* = 0.368, *p* = 0.008) show the most significant correlations with both DST and DSF scores, and remained significant after FDR correction (*p* < 0.05). Volumes of other subfields, including CA1 (DST: *r* = 0.358, *p* = 0.010; DSF: *r* = 0.323, *p* = 0.021), CA4 (DST: *r* = 0.292, *p* = 0.038; DSF: *r* = 0.333, *p* = 0.017), GC-ML-DG (DST: *r* = 0.311, *p* = 0.027; DSF: *r* = 0.328, *p* = 0.019), and hippocampal body (DST: *r* = 0.301, *p* = 0.032; DSF: *r* = 0.312, *p* = 0.026), also show significant correlations with both scale scores, and these results remain significant following FDR correction (*p* < 0.05). Subiculum volume correlates significantly only with DST score (*r* = 0.326, *p* = 0.020), not with DSF score (*p* > 0.05). Notably, CA3 volume shows no significant correlation with either scale score (all *p* > 0.05).

**Table 5:** Correlation between hippocampal subfield volumes and cognitive scale scores in the cancer group.

Hippocampal subregion	DST	DSF
CA1	*r* = 0.358, *p* = 0.010^a^^,^^c^	*r* = 0.323, *p* = 0.021^a^^,^^c^
CA3	*r* = 0.187, *p* = 0.190	*r* = 0.221, *p* = 0.119
CA4	*r* = 0.292, *p* = 0.038^a^^,^^c^	*r* = 0.333, *p* = 0.017^a^^,^^c^
Molecular-layer	*r* = 0.389, *p* = 0.005^b^^,^^c^	*r* = 0.358, *p* = 0.010^a^^,^^c^
GC-ML-DG	*r* = 0.311, *p* = 0.027^a^^,^^c^	*r* = 0.328, *p* = 0.019^a^^,^^c^
Subiculum	*r* = 0.326, *p* = 0.020^a^^,^^c^	*r* = 0.265, *p* = 0.061
Hippocampal body	*r* = 0.301, *p* = 0.032^a^^,^^c^	*r* = 0.312, *p* = 0.026^a^^,^^c^
Hippocampal head	*r* = 0.410, *p* = 0.003^b^^,^^c^	*r* = 0.368, *p* = 0.008^b^^,^^c^

a Indicates *p* < 0.05.

b Indicates *p* < 0.01.

c Indicates *p* < 0.05 after FDR correction.

## 4 Discussion

This study employs neuroimaging techniques in conjunction with standardized neuropsychological assessments to investigate differences in working memory function and hippocampal subfield volumes between middle-aged and older adult cancer patients and healthy control subjects. It further explored the association between hippocampal subfield volumes and working memory.

Inter-group comparisons of cognitive scale results reveal that the cancer group had significantly lower total DST scores and DSF subscores compared to the healthy control group. This finding suggests that the cancer itself or its related treatments may impair working memory. Working memory, a core component of higher cognitive function, refers to the system that temporarily processes and stores information during the execution of cognitive tasks. This theoretical framework, proposed by Baddeley and Hitch in 1974, is now widely applied in cognitive neuroscience research. Our results demonstrated that cancer patients performed significantly worse than healthy controls on the digit span test (DST) and its forward span (DSF) subset. This finding can be interpreted within the multi-component model of working memory proposed by Baddeley and Hitch. The DST, particularly its forward span component, serves as a classical measure for assessing the storage and rehearsal capacity of the phonological loop. Therefore, our study provides direct behavioral evidence for impaired integrity of the phonological loop in cancer patients. Existing evidence indicates that working memory impairment in cancer patients can occur throughout the disease management trajectory, including the pre-treatment baseline period, during treatment, and in long-term follow-up (Anderson et al., 2019; Hardy et al., 2023; Lange et al., 2023). Lange et al. (2016) reported that among older cancer patients, 49% had impairment in at least one cognitive domain, with 25% exhibiting specific working memory impairment. The data obtained in this study align with these previous findings. Zhou et al. (2022) found no significant difference in DST performance between chemotherapy and non-chemotherapy groups in breast cancer patients. Notably, however, their study focused solely on the impact of chemotherapy on cognitive outcomes and did not include a healthy control group to assess the influence of the cancer disease itself.

Neuroimaging analysis further revealed a trend of volume reduction in multiple hippocampal subfields in the cancer group (including CA3, CA4, CA1, hippocampal molecular layer, GC-ML-DG, subiculum, hippocampal head, and hippocampal body). However, only atrophy in the CA3 subfield survived the stringent FDR multiple comparison correction. Daniel et al. (2024), using surface-based morphometry, found significant hippocampal volume reduction after chemotherapy in older breast cancer patients. At the same time, the non-chemotherapy group exhibited atrophy only in the right hippocampus. Notably, an existing fMRI study using a coarse parcellation approach (e.g., anterior/posterior hippocampal dichotomy) to explore properties of different hippocampal regions suggested that chemotherapy particularly induces abnormal functional connectivity in the anterior hippocampus (Feng et al., 2020). Current evidence regarding structural changes in hippocampal subfields in cancer patients is relatively limited. This study achieved fine-grained quantification at the hippocampal subfield level in a population of middle-aged and older adults with cancer. Unlike Liu et al. (2024), who found no group differences in hippocampal subfield volumes in lung cancer patients, this study observed significant atrophy in the CA3 subfield of the cancer group. Considering that gastrointestinal tumors constituted 62.7% of the sample (with 66.7% being stage IV patients), this discrepancy might stem from cancer type heterogeneity. Chronic malnutrition, gut microbiota dysbiosis, and systemic inflammation states characteristic of gastrointestinal tumors could selectively damage the hippocampus via the “gut-brain axis” pathway (Onimus et al., 2024).

Correlation analysis indicates that volumes of the hippocampal molecular layer and hippocampal head are significantly positively correlated with DST scores. This finding provides new evidence for understanding the neural mechanisms by which the hippocampus participates in working memory. According to recent theoretical models of memory systems, the hippocampus may participate in cognitive processing through two mechanisms. The “Standard Consolidation Theory” emphasizes its role in memory consolidation, while the “Indexing Theory” highlights its function as a neural index for cortical memory traces (Su et al., 2024; Liu et al., 2025). Recent studies based on the hippocampus acting as a cortical memory index have demonstrated its involvement in working memory encoding (Daume et al., 2024; Su et al., 2024). The structure-function associations observed here for the molecular layer and hippocampal head are particularly noteworthy. The molecular layer, as the dendritic layer overlaying hippocampal subfields (CA1, CA2, CA3), exhibits plasticity independent of the somatic layer; hence, it is separately segmented in FreeSurfer. Xu et al. (2020) similarly found associations between its volume and longitudinal changes in MMSE and MoCA scores in Parkinson’s disease patients.

The hippocampal head, as the anterior anatomical region of the hippocampal formation, is anatomically tightly connected to the amygdala. Neurodevelopmental studies indicate significant morphological maturation in this region during late childhood (Plachti et al., 2023). A neuroimaging study revealed that patients with semantic dementia exhibit significant left hippocampal atrophy, characterized by a distinct regional selectivity pattern, with prominent atrophy in the anterior subfields and relative sparing of the posterior subfields (Lan et al., 2024). Notably, the “anterior hippocampal subfields” referred to in that study and the “hippocampal head” focused on here have substantial anatomical overlap but are not entirely equivalent. Specifically, the hippocampal head segmented by FreeSurfer has boundaries defined by clear anatomical landmarks, offering high standardization, whereas anterior hippocampal divisions might rely more on researcher-defined manual delineation. Recent research has further expanded understanding of the functional diversity of the hippocampal head, finding its involvement in emotional regulation beyond classical memory functions (Liu et al., 2021). These findings suggest that hippocampal head atrophy may affect cognitive function in cancer patients through a dual mechanism: directly impairing working memory and indirectly interfering with cognition via emotional pathways. Future studies should systematically incorporate depression and anxiety scales (e.g., HAMD, HAMA) to quantify the influence of emotional factors in hippocampal-mediated cognitive impairment. Notably, the significantly atrophied CA3 subfield showed no significant correlation with either test, possibly reflecting its functional specificity. A basic research study found that chemotherapy decreased PTPRO expression in the CA3 region of female mice, and PTPRO knockout mice exhibited deficits in the Y-maze (Yao et al., 2023). Such outcomes suggest the CA3 region may preferentially regulate spatial working memory rather than verbal working memory.

This study identified associations between specific hippocampal subfield atrophy and working memory impairment in middle-aged and older adult cancer patients, providing neuroimaging evidence for the neural mechanisms of cancer-related cognitive impairment. Nevertheless, several limitations warrant attention: First, due to the cross-sectional design, we cannot determine causality between cognitive differences and volumetric changes. Second, the structural analysis in this study focused exclusively on gray matter volume. As growing evidence from research on schizophrenia, autism spectrum disorder, and depression has demonstrated, gray matter volume and gray matter concentration/density are complementary measures that can reveal distinct aspects of brain atrophy (Rootes-Murdy et al., 2021; Liloia et al., 2024). Our study did not incorporate an analysis of gray matter concentration, which may have limited a more comprehensive understanding of cancer-related structural alteration patterns. Third, the sample size was relatively limited, and although the sample included middle-aged and older adults, no stratified comparisons were performed across different age groups. Additionally, while emotional state may influence cognitive performance, this study did not systematically assess it, which may somewhat affect the interpretation of the results.

Future research should aim to expand sample sizes through multi-center collaborations, which would not only help validate the generalizability of the current findings but also enable deeper investigation into potential age-related heterogeneity in the neural mechanisms of CRCI. Methodologically, longitudinal designs are recommended, combined with multimodal neuroimaging techniques and molecular biomarker analysis. Structural neuroimaging assessments should integrate complementary measures such as gray matter volume and gray matter concentration. Such an approach would allow for a more systematic elucidation of the specific pathways linking different cancer types and treatment modalities to hippocampal subfield damage, ultimately providing more precise evidence for uncovering the neuropathological mechanisms of CRCI.

## Data Availability

The original contributions presented in the study are included in the article/supplementary material, further inquiries can be directed to the corresponding author.
